# Highly invasive and poorly differentiated corneal squamous cell carcinoma in a dog

**DOI:** 10.1186/s12917-019-1790-3

**Published:** 2019-02-07

**Authors:** López-Murcia María del Mar, Mayordomo-Febrer Aloma, Viana David, Mozos Elena, Ortega Joaquín

**Affiliations:** 1 Departamento de Medicina y Cirugía Animal, Facultad de Veterinaria, Universidad Cardenal Herrera-CEU, CEU Universities, Tirant lo Blanc, 7, 46115 Alfara del Patriarca, Valencia Spain; 2 Departamento de Producción y Sanidad Animal, Salud Pública Veterinaria y Ciencia y Tecnología de los alimentos, Unidad de Histología y Anatomía Patológica, Facultad de Veterinaria, Universidad Cardenal Herrera-CEU, CEU Universities, Tirant lo Blanc, 7, 46115 Alfara del Patriarca, Valencia Spain; 3 Departamento de A. y Anatomía Patológica Comparadas, Facultad de Veterinaria, Ed. Sanidad Animal. Ctra, Universidad de Córdoba, Campus de Rabanales, Madrid-Cádiz Km 396A, 14014 Córdoba, Spain

**Keywords:** Primary corneal squamous cell carcinoma, Dog, Corneal neoplasia

## Abstract

**Background:**

Primary corneal tumors are unusual in dogs although there has been a rise in the prevalence of canine corneal squamous cell carcinoma in the past decades due to different factors. Exposure to ultraviolet radiation, presence of chronic keratitis or history of superficial trauma are some of them. We report for the first time a highly infiltrative corneal neoplasia with both exophytic and deep stromal growth, which presented atypical histologic features of a squamous cell carcinoma.

**Case presentation:**

An adult male French bulldog was referred with an exophytic, pink to white gelatinous mass occupying approximately 70% of the central cornea on the right eye. Histological findings from the excisional biopsy were consistent with corneal carcinoma and transconjunctival enucleation was performed at the request of the owner. A final diagnosis of primary corneal squamous cell carcinoma was done based on the squamous differentiation observed in the neoplastic cells of the superficial layers. However, cells in deeper layers were less differentiated, showed pseudoacinar formations and did not expressed marker for stratified squamous epithelium (i.e., cytokeratin 5/6). The dramatic thickening of the cornea and the fact of observing neoplastic cells almost at the level of the Descemet’s membrane make this case very unusual as the squamous cell carcinoma in dogs tends to involve the superficial stroma or colonize the corneal surface as an exophytic lesion.

**Conclusions:**

Based on the histological findings, a high infiltrative and poorly differentiated corneal squamous cell carcinoma was diagnosed. In terms of clinical relevance, our results suggest that corneal lesions compatible with neoplasia need an early diagnosis in order to prevent the aggressive growth of the tumor and the enucleation of the eye.

## Background

Primary corneal neoplasia is rare in dogs, although a few cases of squamous cell carcinoma (SCC) have been already described [1–5].

Some factors seem to be involved in the development of the corneal SCC, such exposure to ultraviolet radiation, chronic keratitis or superficial trauma [2, 4]. Brachycephalic breeds are also overrepresented [2].

Corneal SCC has histologic features similar to those in other locations [6]. In the few published case reports of this tumor in dogs, most occurred at the corneoscleral limbus or the ocular adnexa. In other cases there is no involvement of the limbus, suggesting the tumors originate from the corneal epithelium itself. The mean age of dogs at the time of diagnosis was 9.6 years [2].

In dogs, the tumor involves the superficial stroma or it colonize the corneal surface as an exophytic lesion [1, 3–5, 7–10]. However, in other species the tumor can be very infiltrative. In this way, a corneal stromal-invasive SCC has been described recently in horses, as an unusual variant of ocular and adnexal SCC [11]. In cattle, the tumor can infiltrate the anterior chamber and eventually the entire globe [12].

We describe and characterize the clinical appearance and histopathological findings of a central primary corneal squamous cell carcinoma that grew into the deep stroma, almost reaching the Descemet’s membrane, over a short period in a 7 year-old male French bulldog. To the best of authors’ knowledge, this is the first report of a deep corneal stromal invasive carcinoma in a dog.

## Case presentation

A 7-year-old male French bulldog was referred to the Veterinary Teaching Hospital at CEU Cardenal Herrera University for a corneal mass on the right eye that had been enlarging over a 2-month period. The dog had a complete excision of the third eyelid gland 5 years before and developed an iatrogenic keratoconjunctivitis sicca that was being controlled with topical compounded 1% cyclosporine eye drops every 24 h and artificial tears every 8 h.

**Fig. 1:**
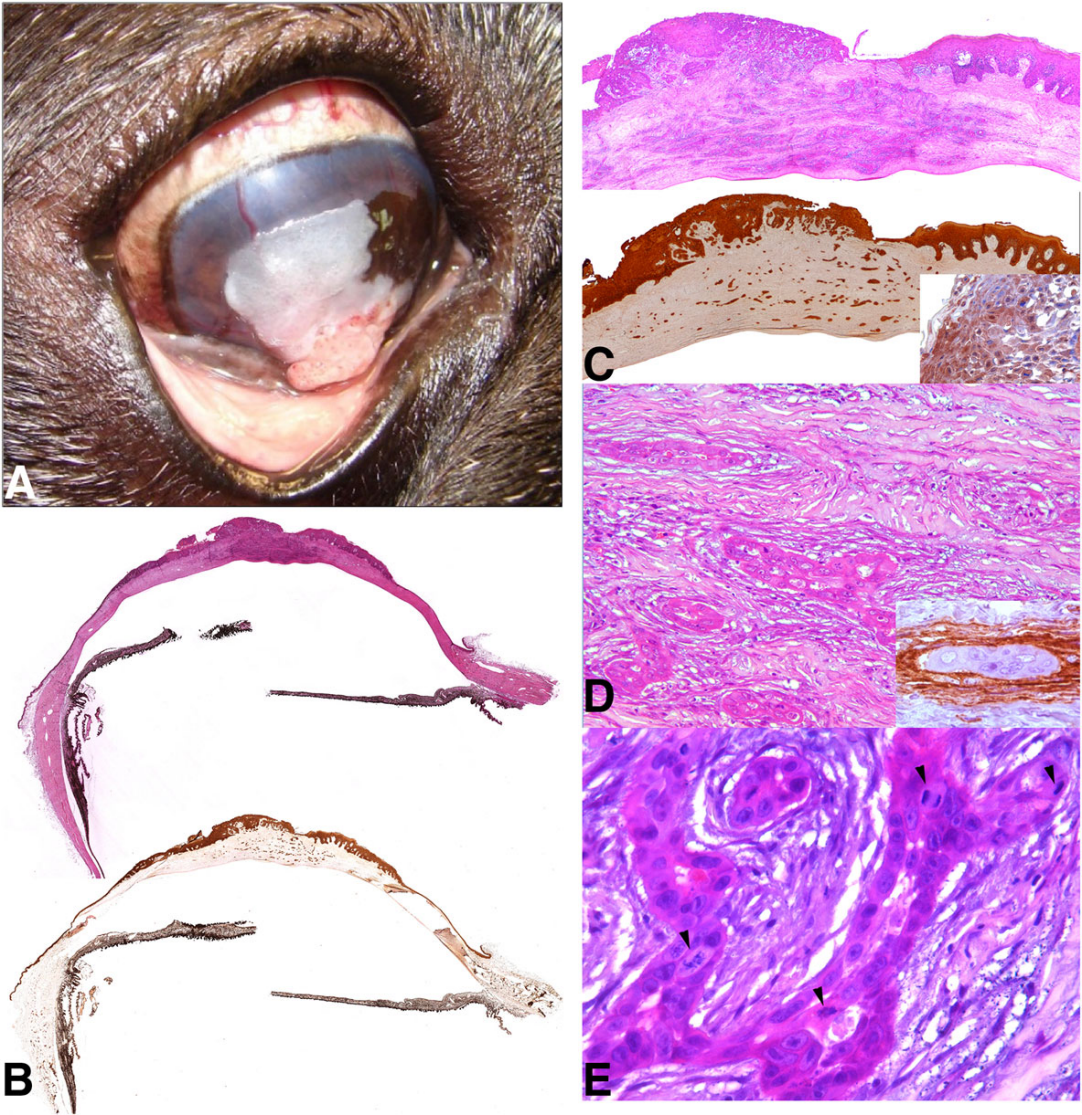
**a** Exophytic white irregular mass occupying 70% of the central cornea. **b** and **c** Subgross photography of partial eye and cornea respectively (H-E and Cytokeratin). Neoplastic cells produce epithelium disruption and deep corneal stromal invasion. Inset: Detail of the neoplastic corneal epithelium reacted with the CK5. **d** A proliferation of epithelial cells arranged in tubules was extending throughout the substantia propria of the cornea. Inset: Detail of the tubular structures surrounded by cells positive to muscle actin antibody (myofibroblasts). **e** Neoplastic cells arranged in tubules with prominent nucleoli, anisokaryosis and anisocytosis. The mitotic figures were moderate (arrowhead) H-E

Initial ophthalmic examination revealed an exophytic pink to white gelatinous mass occupying approximately 70% of the central cornea on the right eye (Fig. 1a). Dazzle reflex was inconstant whereas menace response was negative. Fluorescein staining was negative. Slit lamp biomicroscopy examination (Kowa® SL-14, Kowa Company, Tokyo, Japan) showed severe increase of the corneal thickness, pigment deposit and superficial vascularization; the mass was infiltrating the corneal stroma. The rest of the ocular examination including Schirmer tear test-1 (19 mm/min, Sno-Strips®. Chauvin Pharmaceuticals Ltd. Essex, Great Britain) and intraocular pressure obtained by applanation tonometry (13 mmHg, Tonopen XL®, Mentor, Norwell, MA, USA) was within the normal limits. Indirect ophthalmoscopy could not be performed. The ophthalmic exam of the left eye was unremarkable, and no abnormalities were observed on physical examination.

At that time, the temptative diagnosis for the right eye was a corneal neoplasia; other differential diagnoses were granulation tissue and chronic inflammatory process. An excisional superficial keratectomy was performed under general anesthesia as previously described [13]. Histological findings were consistent with corneal carcinoma and transconjunctival enucleation was finally performed. The globe, conjunctiva and nictitant membrane were fixed intact in 10% neutral buffered formalin and submitted for histopathologic examination.

Locally expansive and infiltrative masses were observed arising from the corneal epithelium and extended into the deeper layers of the corneal stroma (Fig. 1b-d). Neoplastic cells were polyhedral with abundant eosinophilic cytoplasm and evident intercellular bridges and arranged in nest, cords or pseudoacini. These cells had large euchromatic nuclei with single prominent nucleoli. Abundant dyskeratotic foci (squamous differentiation) were observed in the neoplasia, but no keratin pearls were present. Anisokaryosis and anisocytosis were moderate, and the mitotic index was high (3–5 mitosis by high power field, Fig. 1e). An immunohistochemical panel was performed in order to better characterize the neoplasia: monoclonal mouse anti-human antibodies against pan-cytokeratin (clones AE1/AE3, “ready-to-use”), cytokeratin 5/6 (clone D5/16 B4; dilution: 1/50), cytokeratin 7 (clone OV-TL 12/30; dilution: 1/50) and muscle actin (clone HHF35, “ready-to-use”) from DAKO, Glostrup, Denmark. All neoplastic cells were strongly positive to AE1/AE3 pan-cytokeratin antibody (Fig. 1c), but only cells within the neoplastic corneal epithelium reacted with the CK5/6 (marker used for stratified epithelium, Fig. 1c). However, those cells located in the deeper layers of the corneal stroma and arranged as pseudoacini were both negative for CK5/6 and CK7 (specific marker for epithelial cell from glandular origin) and surrounded by elongated cells positive to muscle actin antibody (myofibroblasts cells, Fig. 1d). Neoplastic cells were near Descemet’s membrane but it was not invaded. Occasional blood vessels and fibroplasia were observed near the neoplastic foci. The corneal epithelium adjacent to the neoplasia was thickened and dysplastic. A mild inflammatory cell infiltrate, composed of a mixed population of lymphocytes and neutrophils, was also present at the tumor site. The stroma adjacent to the neoplastic tissue showed numerous melanin-laden macrophages. Based on the histological findings, a diagnosis of a poorly differentiated squamous cell carcinoma with deep stromal invasion was made.

## Discussion and conclusions

Primary corneal neoplasia is rare in dogs; reports include papilloma, limbal melanocytoma, hemangioma, hemangiosarcoma, lymphoma and melanocytoma [14, 15]. Corneal SCC is uncommon in dogs compared with other species, such cattle or horses [6, 12], even though there has been a rise in the prevalence of canine SCC in the past decades, due to different factors.

In one hand, brachycephalic dogs are increasing in popularity, being a breed related with a high number of ocular abnormalities, including chronic corneal inflammation. In this way, a retrospective study showed corneal SCC overrepresented in brachycephalic breeds (77%) [2]. On the other hand, a potential association between the use of immunosuppressive drugs and corneal SCC has also been suggested [2, 5, 8]. Other factors involved in the development of this tumor include ultraviolet radiation exposure, chronic keratitis or superficial trauma [1, 4]. All of these features are present in our case, a French bulldog with chronic superficial keratitis and treatment with topical immunosuppressive therapy over a 5-year period.

The differential diagnoses, based on the clinical presentation, were corneal neoplasia, exacerbation of a chronic inflammatory process or granulation tissue secondary to corneal trauma. Histological examination of biopsy samples is the most accurate way to diagnose neoplasia. An excisional superficial keratectomy was performed and a diagnosis of malignant neoplasm of epithelial origin was initially made. Interestingly, after enucleation, histological features observed in the cornea were consistent with an atypical SCC. Neoplastic cells found in the stroma did not form solid nests of epithelial cells with multiple concentric layers of keratin (“keratin perls”, expected for a SCC), but they were arranged mostly in a pseudoacinar pattern and surrounded by myoepithelial-like cells (which is more consistent with adenocarcinoma than SCC). However, as there are no glands present within the cornea and there was no evidence of previous glandular neoplasia in the surrounding tissues (or anywhere in the dog) which could cause metastasis to cornea, nor indication of dermoids or any other congenital abnormality in the cornea, the chances to have a corneal adenocarcinoma were very low. One possibility was that cells from corneal epithelium have undergone more undifferentiated and pleomorphic acquiring a glandular appearance, as the transition from SCC to adenocarcinoma has been previously described in other epithelial tumor in humans [16]. Specific immunohistochemical markers for epithelial tumors were used in order to better understand the nature of this unusual neoplasia. Superficial neoplastic cells from corneal epithelium were positive for CK5/6 but deeper acinar formations were negative for both CK5/6 and CK7. We suspect that neoplastic cells were losing specific cytokeratins when they rearranged their cytoskeleton to migrate into the deeper layers of the corneal stroma and became more undifferentiated. This unusual histological feature maybe related to the very invasive behavior of this tumor compare to most of the SCCs previously reported.

The prognosis for survival of the patients with corneal SCC is relatively good [6] since the neoplasia appears to have low metastatic activity. However, the prognosis for globe maintenance varies. Therefore, the early recognition of the lesion, the diagnosis and the promptness treatment is of most importance for clinicians. Follow up every 6 months in our case did not reveal any sign of orbital recurrence or metastatic presentation, 2 years after the surgery.

Treatment of corneal neoplasia depends on tumor size, location, availability of equipment and economic factors. According to most authors, surgery is the first choice treatment for corneal SCC, alone or with adjunctive therapy [3]. Superficial keratectomy has been used successfully in SCC in dogs, as facilitates complete removal of the abnormal appearing tissue [5, 7–9]. Adjuvant therapies have been found to improve clinical outcomes and include nitrous oxide cryotherapy, beta-irradiation, chemotherapeutic methods as topical mitomicine C or 5-fluorouracil and plesiotherapy [3, 4, 7, 10]. Topical 1% 5-fluorouracil ointment as a sole therapy has been used successfully in very small corneal SCC [1]. In our case, conservative treatment options were very limited, and the depth of the lesion into the corneal stroma and the wide tumor extension were determinants for the enucleation. A complete lamellar keratoplasty could be performed, although the owner declined any risk therapeutic option.

In all the published clinical cases of canine corneal SCC, the tumor appeared either as an exophytic lesion affecting mainly the corneal epithelium, or grew by invasion of the superficial corneal stroma [2]. In horses, SCC is the most common neoplasm of the eye, affecting the nictitant membrane, nasal canthus, limbus, cornea and eyelids [17]. Ocular SCC originates from the cornea, conjunctiva, or limbus, with the lateral limbus being the most usual location [18]. Most commonly appears as a nodular, elevated, white-pink mass; however a corneal stromal invasive SCC has been described, showing an unusual and distinctive infiltrative growth pattern with a smooth and intact anterior corneal epithelium and Descemet’s membrane. In this case, the cornea was markedly thickened, neovascularized and edematous [11, 17]. When originates at the limbus, it has been previously described that the tumor can spread deeply around the border of Descemet’s membrane, infiltrating even the uveoscleral meshwork and iridocorneal angle [19]. The case reported here shows a very invasive neoplasia with both exophytic and deep stromal growth, as the tumor almost reached the Descemet’s membrane, causing a dramatic thickened of the entire cornea over a short period in a dog.

To the authors’ knowledge, this is the first report of an atypical canine corneal squamous cell carcinoma with deep stromal invasion. In terms of clinical relevance, our results suggest that a prompt diagnosis should be performed, in order to prevent the growth of the tumor. A misdiagnosis can potentially delay treatment conservative options.

## Abbreviation

SCC: Squamous cell carcinoma
